# Metabolomic profiling of glucose homeostasis in African Americans: the Insulin Resistance Atherosclerosis Family Study (IRAS-FS)

**DOI:** 10.1007/s11306-023-01984-1

**Published:** 2023-04-02

**Authors:** Hayrettin Okut, Yingchang Lu, Nicholette D. Palmer, Yii-Der Ida Chen, Kent D. Taylor, Jill M. Norris, Carlos Lorenzo, Jerome I. Rotter, Carl D. Langefeld, Lynne E. Wagenknecht, Donald W. Bowden, Maggie C. Y. Ng

**Affiliations:** 1 Center for Precision Medicine, Wake Forest School of Medicine, Winston-Salem, NC USA; 2 Department of Population Health, University of Kansas School of Medicine-Wichita, Wichita, KS USA; 3 Division of Genetic Medicine, Vanderbilt Genetics Institute, Vanderbilt University Medical Center, Nashville, TN 37232 USA; 4 Department of Biochemistry, Wake Forest School of Medicine, Winston-Salem, NC USA; 5 Department of Pediatrics, The Institute for Translational Genomics and Population Sciences, The Lundquist Institute for Biomedical Innovation at Harbor-UCLA Medical Center, Torrance, CA USA; 6 Departments of Epidemiology, Colorado School of Public Health, University of Colorado Denver, Aurora, CO USA; 7 Department of Medicine, University of Texas Health Science Center, San Antonio, TX USA; 8 Department of Biostatistical Sciences, Wake Forest School of Medicine, Winston-Salem, NC USA; 9 Division of Public Health Sciences, Wake Forest School of Medicine, Winston-Salem, NC USA; 10 Department of Internal Medicine, Wake Forest School of Medicine, Winston-Salem, NC USA

**Keywords:** Metabolomics, Glucose homeostasis, African Americans

## Abstract

**Introduction:**

African Americans are at increased risk for type 2 diabetes.

**Objectives:**

This work aimed to examine metabolomic signature of glucose homeostasis in African Americans.

**Methods:**

We used an untargeted liquid chromatography-mass spectrometry metabolomic approach to comprehensively profile 727 plasma metabolites among 571 African Americans from the Insulin Resistance Atherosclerosis Family Study (IRAS-FS) and investigate the associations between these metabolites and both the dynamic (S_I_, insulin sensitivity; AIR, acute insulin response; DI, disposition index; and S_G_, glucose effectiveness) and basal (HOMA-IR and HOMA-B) measures of glucose homeostasis using univariate and regularized regression models. We also compared the results with our previous findings in the IRAS-FS Mexican Americans.

**Results:**

We confirmed increased plasma metabolite levels of branched-chain amino acids and their metabolic derivatives, 2-aminoadipate, 2-hydroxybutyrate, glutamate, arginine and its metabolic derivatives, carbohydrate metabolites, and medium- and long-chain fatty acids were associated with insulin resistance, while increased plasma metabolite levels in the glycine, serine and threonine metabolic pathway were associated with insulin sensitivity. We also observed a differential ancestral effect of glutamate on glucose homeostasis with significantly stronger effects observed in African Americans than those previously observed in Mexican Americans.

**Conclusion:**

We extended the observations that metabolites are useful biomarkers in the identification of prediabetes in individuals at risk of type 2 diabetes in African Americans. We revealed, for the first time, differential ancestral effect of certain metabolites (i.e., glutamate) on glucose homeostasis traits. Our study highlights the need for additional comprehensive metabolomic studies in well-characterized multiethnic cohorts.

**Supplementary Information:**

The online version contains supplementary material available at 10.1007/s11306-023-01984-1.

## Introduction

Metabolomics, the comprehensive profiling of small molecule metabolites in biological systems, has proven to be a powerful tool for understanding biochemical pathways and disease mechanisms (Newgard, 2017). High-throughput metabolomic profiling technologies using mass spectrometry and nuclear magnetic resonance can identify thousands of metabolites simultaneously. These technological advances have led to the successful application of metabolomics to identify new biomarkers for cardiometabolic diseases and improve our mechanistic understanding of these diseases and related traits (Newgard, 2017).

Type 2 diabetes is often preceded by a long period of prediabetes, characterized by insulin resistance and impaired insulin secretion. Previous studies have demonstrated associations between a wide-range of circulating metabolites and insulin resistance, insulin secretion and type 2 diabetes risk primarily in populations of European ancestry (Guasch-Ferre et al., 2016; Newgard, 2017). Increased levels of branched-chain amino acids (BCAA: leucine, isoleucine and valine), aromatic amino acids (phenylalanine and tyrosine), glutamate, lysine and their metabolites, but reduced levels of glutamine and glycine are associated with insulin resistance, impaired insulin secretion and/or type 2 diabetes risk (Cheng et al., 2012; Davalli et al., 2012; Ferrannini et al., 2013; Floegel et al., 2013; Gall et al., 2010; Guasch-Ferre et al., 2016; Huffman et al., 2009; Liu et al., 2019; Menni et al., 2013; Newgard, 2017; Newgard et al., 2009; Shah et al., 2012; Stancakova et al., 2012; Thalacker-Mercer et al., 2014; Vangipurapu et al., 2019; Walford et al., 2016; Wang et al., 2011, 2013; Wurtz et al., 2012a, 2012b, 2013). Several carbohydrate metabolites, including glucose, fructose and inositol, are hallmarks for type 2 diabetes (Drogan et al., 2015; Floegel et al., 2013; Guasch-Ferre et al., 2016; Padberg et al. 2014; Wurtz et al., 2012a, 2012b). In addition, lipid subclasses such as phospholipids, sphingomyelins and triglycerides, are associated with risk of prediabetes and type 2 diabetes (Ferrannini et al., 2013; Floegel et al., 2013; Guasch-Ferre et al., 2016; Rhee et al., 2011; Wang-Sattler et al., 2012). Few studies including non-European and/or multi-ethnic populations have been reported. Many of the amino acids are replicated for association with insulin resistance and type 2 diabetes risk in Asians (Chen et al., 2016, 2019; Tai et al., 2010). Similarly, several amino acids, lipids and carbohydrate metabolites are associated with insulin resistance, incident and prevalent type 2 diabetes in African Americans, Mexican Americans and multi-ethnic populations(Chen et al., 2022; Lee et al., 2016; Palmer et al., 2015, 2018; Rebholz et al., 2018). Large-scale multi-ethnic studies such as The Consortium of Metabolomics Studies (COMETS) are ongoing with available fasting glucose and insulin levels (Yu et al., 2019).

African Americans are at increased risk of obesity, type 2 diabetes and other cardiometabolic diseases. However, few large-scale metabolomic studies have examined glucose homeostasis in this population, particularly for the sophisticated dynamic measures. In this study, we used an untargeted liquid chromatography-mass spectrometry (MS) metabolomic approach to examine plasma metabolites among 571 African Americans from the Insulin Resistance Atherosclerosis Family Study (IRAS-FS) and investigate the associations between these metabolites and both the basal and dynamic measures of glucose homeostasis traits. We also compared our results in African Americans with our previous results in IRAS-FS Mexican Americans (Palmer et al., 2018).

## Methods

### Study participants

Study participants were recruited from the Insulin Resistance Atherosclerosis Family Study (IRAS-FS) (Henkin et al., 2003; Palmer et al., 2018). This study included 42 African American families from Los Angeles, CA. These families were recruited based on large family size, irrespective of disease status. A total of 572 African Americans without diabetes were examined for association between metabolites and glucose homeostasis traits. A similar metabolomics study in Mexican Americans from the IRAS-FS has been previously reported (Palmer et al., 2018). The study protocol was approved by the institutional review board of the clinical and analysis sites. All participants provided their written informed consent.

### Glucose homeostasis measurements

Insulin sensitivity index (S_I_) and glucose effectiveness (S_G_) were calculated from the frequently sampled intravenous glucose tolerance test (FSIGT) using mathematical modeling method (MINMOD version 3.0; Harms Software, CA) (Pacini & Bergman, 1986). MINMOD uses FSIGT glucose and insulin data to fit two mathematical models for glucose disappearance and insulin kinetics, respectively.

Glucose disappearance is calculated as:

1 $$\frac{dG}{{dt}} = - \left( {p_{1} + X\left( t \right)} \right) \cdot G\left( t \right) + p_{1} \cdot G_{b} ,\quad G\left( 0 \right) = G_{0}$$

2 $$\frac{dX}{{dt}} = - p_{2} \cdot X\left( t \right) + p_{3} \cdot \left( {I\left( t \right) - I_{b} } \right),\quad X\left( 0 \right) = 0$$

Insulin kinetics is calculated as:

3 $$\frac{dI}{{dt}} = - n \cdot I\left( t \right) + \gamma \cdot \left( {G\left( t \right) - h} \right) \cdot t,\quad I\left( 0 \right) = I_{0}$$

*G(t)* and *I(t)* represent the time courses of glucose and insulin in plasma following glucose injection. *G*_*b*_ and *I*_*b*_ are basal values. *X(t)* describes the insulin effect on net glucose disappearance and *p*_*1*_, *p*_*2*_, *p*_*3*_, *G*_*0*_, *n*, *γ*, *h* and *I*_*0*_ are parameters. Parameter *p*_*1*_ represents S_G_ which is the effect of glucose per se at basal insulin to normalize glucose concentration within the extracellular glucose pool. The ratio between *p*_*3*_ and *p*_*2*_ represents S_I_ which is the insulin dependent increase in the net glucose disappearance rate. Acute insulin response to glucose (AIR) was calculated as the increase in insulin concentrations at 2 to 8 min above the basal (fasting) insulin level after a bolus glucose injection at 0 to 1 min. AIR is a measure of pancreatic function and insulin release. Disposition index (DI) was calculated as AIR multiplied by S_I_, which reflects the ability of the body to compensate for changes in either β-cell function or peripheral insulin action. Basal measures included the updated homeostatic model assessment of insulin resistance (HOMA-IR) and beta cell function (HOMA-B) (Levy et al., 1998).

### Metabolite measurements

Metabolite profiling was performed on stored (at -80 °C) fasting plasma samples collected at the 1999–2002 baseline survey. Metabolite detection and quantification was conducted by Metabolon, Inc. (Durham, North Carolina) using untargeted liquid chromatography-mass spectroscopy (MS) (DiscoveryHD4 panel). Samples were prepared using the automated MicroLab STAR system (Hamilton Company, Salt Lake City, UT). A methanol extraction was used to remove protein, dissociate small molecules bound to protein or trapped in the precipitated protein matrix, and to recover chemically diverse metabolites. The resulting extract was divided into five fractions: two for analysis by two separate reverse phase/ultra-performance liquid chromatography-MS/MS methods with positive ion mode electrospray ionization (ESI), one for analysis by reverse phase/ultra-performance liquid chromatography-MS/MS with negative ion mode ESI, one for analysis by hydrophilic interaction liquid chromatography/ultra-performance liquid chromatography-MS/MS with negative ion mode ESI and one sample was reserved for backup. All methods utilized a Waters ACQUITY ultra-performance liquid chromatography (UPLC) and a Thermo Scientific Q-Exactive high resolution/accurate mass spectrometer interfaced with a heated electrospray ionization source and Orbitrap mass analyzer operated at 35,000 mass resolution. Raw data were extracted, peak-identified and quality control processed using Metabolon’s hardware and software. Compounds were identified by comparison to library entries of purified standards or recurrent unknown entities. Peaks were quantified using area under the curve. Several types of controls were analyzed in addition to experimental samples: a technical replicate, pooled matrix sample generated from a small volume of each experimental sample; process blanks, extracted water samples; and QC standards, a cocktail of QC standards chosen not to interfere with the measurement of endogenous compounds were spiked into every analyzed sample, allowed instrument performance monitoring and aided chromatographic alignment. This panel identified and provided relative quantification of known chemical compounds among amino acid, carbohydrate, energy, lipid, nucleotide, and peptide super pathways. In addition to individual named biochemicals; super- and sub-pathways were annotated based on a combination of pathway and chemical structure similarities to serve as a guide for interpretation. Prior to return, data were block corrected for a run day, normalized by batch, and volume extracted. Missing data for metabolites were imputed to the minimum value for the respective metabolite. Each metabolite in original scale was rescaled to set the median equal to one. Since the metabolite distributions were highly skewed, metabolite values were winsorized at 1% and 99% to reduce the impact of outliers on parameter estimation. Among 572 African Americans, one individual had 132 metabolite outliers (± 4 SD) and was excluded from downstream analyses. In addition, six metabolites with > 50% missing values were excluded. A total of 727 out of 733 metabolites passed the quality control and were retained for the final analyses (Supplementary Table 1).

### Univariate metabolite analysis

Phenotype variables were transformed to approximate normal distributions as follows: S_I_, HOMA-IR and HOMA-B were natural log transformed; AIR and DI were square-root transformed but retained the mathematical sign of the original value; S_G_ was not transformed. General linear mixed model was used to test for the association between each metabolite and glucose homeostasis traits after adjusting for age, sex, BMI and pedigree structure in IRAS-FS. The restricted maximum likelihood method in PROC MIXED in SAS was used to fit for each glucose homeostasis trait. The proportion of variance (R^2^_adj_) explained by significantly associated metabolites from univariate metabolite models for a given glucose homeostasis trait was estimated using residual values of mixed model fitting after conditioning on covariates. To control for multiple tests, the Bonferroni-corrected *P* value of 6.8 × 10^–5^ (0.05/727 annotated metabolites) was used as the threshold for statistical significance.

### Metabolite selection model

The metabolite levels within each metabolic pathway (Supplementary Table 1) are potentially correlated with each other. We used the scaled elastic net regularized regression method to disentangle the linear dependency of correlated metabolites in the prediction of glucose homeostasis traits. In the scaled elastic net regression model, the LASSO penalty function (*L*_*1*_) and ridge penalty function (*L*_*2*_) shrink the estimates of the regression coefficients towards zero relative to the maximum likelihood estimates. This shrinkage prevents overfitting arose from either collinearity of the metabolites or high-dimensionality of data when *p* >  > *n* (where *p* is the number of metabolites, and *n* is the number of individuals). In the procedure of regularization with an elastic net, the coefficient of ridge regression is estimated, then a LASSO algorithm is performed on the ridge regression coefficient to shrink the coefficient. The ridge regression penalty function (*L*_*2*_) penalizes the square of the regression coefficients for the predictors, shrinking coefficients from correlated predictors proportionally toward zero. The LASSO penalty (*L*_*1*_) imposes a penalty on the absolute value of the coefficients, shrinking coefficients by a constant factor, and can select a subset of predictors by shrinking coefficients for the least predictive predictors exactly to zero. The scaled elastic net uses these penalties to minimize the objective function in Eq. 4.

4 $$\mathop {\mathop {\arg \min }\limits_{{\beta_{0} ,\beta_{j} }} \left[ {\sum\nolimits_{i = 1}^{n} {\left( {y_{i}^{*} - \beta_{0} - \sum\nolimits_{j = 1}^{p} {\beta_{j} X_{ij} } } \right)^{2} + \lambda } \sum\nolimits_{j = 1}^{p} {\left( {\frac{1 - \alpha }{2}\beta_{j}^{2} + \alpha |\beta_{j} |} \right)} } \right]}\limits_{{}}$$

where *n* is the number of observations (*i* = 1, 2, …, *n*), $y_{i}^{*}$ is the *n* × 1 vector of residual values of glucose homeostasis traits after adjusting for age, sex, BMI and pedigree structure with the mixed model, *p* (*j* = 1, 2, …, *p*) is the number of metabolites, *β*_0_ and *β* = (*β*_1_,* β*_2_,…..,* β*_*p*_)^*T*^ are the fitting parameters for intercept (known as bias) and metabolites, *X*_*ij*_ is the *n x p* matrix standardized metabolites, 0 ≤ *α* ≤ 1 is the tuning parameter that controls the balance between the LASSO (*α* = 1) and ridge (*α* = 0) penalties, *λ* ≥ 0 is positive regularization (or penalty) parameter with the degree of shrinkage increasing as *λ* increases for a given *α* value (Goeman et al. 2014). The adjusted R^2^ given in Eq. (2) was used as the stopping criteria.

5 $$\overline{R}^{2} = 1 - \frac{{\left( {n - i} \right)\left( {1 - R^{2} } \right)}}{n - p}$$

where* i* is equal to 1 if there is an intercept and 0 otherwise, *n *is the number of observations used to fit the model, and *p* is the number of parameters needed to be estimated in the model.

For the internal validation of the scaled elastic net model, 2000 bootstrapping were conducted by using the model-average approach. As the initial round of model average approach using bootstrapping contains many effects, we used the refit option to obtain a more parsimonious model for better generalization. Thus, for each data sample in the refit, a least squares model was fit with no effect selection (only intercept in the model). After 2000 bootstrapping of which 90% of each sample is used as training data, the number of non-zero, non-zero percentage and estimated quantiles (25%, 50% and 75%) information for intercept and parameter estimates were used. Metabolites that were selected for more than 20% of samples after 2000 bootstrapping were reported for the metabolite selection model. Before running model-average, a log scale grid search algorithm was used to specify the optimal ridge regression parameter, *L*_*2*_ for the scaled elastic net. The predicted values ${\widehat{y}}^{(i)}$ for average model *i* are given by

$${\widehat{y}}^{(i)}={\varvec{X}}{{\varvec{\beta}}}^{({\varvec{i}})}$$

where **X** is the design matrix of the data to be scored. Averages are obtained as:

6 $$\hat{y}^{\left( * \right)} = X\left( {\frac{1}{N}\mathop \sum \limits_{i = 1}^{N} \beta^{\left( i \right)} } \right) = X\beta^{\left( * \right)}$$

where parameter *j,* ${\beta }_{j}^{(*)}=\frac{1}{N}{\sum }_{i=1}^{N}{\beta }_{j}^{(i)}$(SAS Inst., 2021). All statistical analyses were performed using SAS (SAS Inst, 2016) and the statistical R package, version 3.3.3 (http://www.r-project.org/).

### Metabolite effect size comparisons for glucose homeostasis traits between African Americans and Mexican Americans

Identical analytical protocols were implemented for data processing in both African Americans and Mexican Americans in IRAS-FS, i.e., metabolite data quality control, outcome transformation and covariate adjustments. The difference in metabolite effect sizes between African Americans and Mexican Americans were tested using the Z statistic. To control for multiple tests, the Bonferroni corrected *P* value of 6.8 × 10^–5^ (0.05/727 annotated metabolites) was used as the threshold for statistical significance. The metabolites that showed significant associations with glucose homeostasis traits in both populations were further characterized.

## Results

### Characteristics of IRAS-FS

This study included 571 IRAS-FS African Americans with a mean (± SD) age of 42.3 ± 13.7 years (Table 1). Overall, study subjects comprised of more women (59.2%) and were, on average, overweight (BMI = 30.0 ± 6.8 kg/m^2^). Participants had a mean S_I_ of 1.64 ± 1.17 × 10^–4^ min/μU/mL, mean AIR of 1013 ± 830 μU/mL/min, and a resulting mean DI of 1436 ± 1269. In comparison with IRAS-FS Mexican Americans (Supplementary Table 2) (Palmer et al., 2018), African Americans were more insulin resistant based on the dynamic glucose homeostasis measures (i.e., reduced S_I_); however, African Americans had reduced levels of both HOMA-IR and HOMA-B when compared with Mexican Americans (Supplementary Table 2). Most pairwise correlations (except between DI and HOMA-B) among the six glucose homeostasis traits in African Americans were significant with absolute correlation coefficients ranging from 0.16 to 0.84. AIR was more correlated with DI and S_G_ (*r*_S_ = 0.62 and 0.30, respectively) in IRAS-FS African Americans than in IRAS-FS Mexican Americans (*r*_S_ = 0.50 and 0.20, respectively); however, AIR is less correlated with S_I_ and HOMA-B (*r*_S_ = -0.16 and 0.18, respectively) in IRAS-FS African Americans than in IRAS-FS Mexican Americans (*r*_S_ = -0.27 and 0.35, respectively; Supplementary Table 3).

**Table 1:** Clinical characteristics of the IRAS-FS African American cohort (N_max_ = 571)

Demographics	Mean ± SD	Interquartile range
Age, year	42.3 ± 13.7	(31.4–51.9)
Women, %	59.2	
BMI, kg/m^2^	30.0 ± 6.8	(25.1–34.7)
Dynamic measures		
S_I_, × 10^–4^ min/μU/mL	1.64 ± 1.17	(0.8–2.31)
S_G_, per min	0.021 ± 0.008	(0.016–0.025)
AIR, μU/mL/min	1013 ± 830	(427–1326)
DI	1436 ± 1269	(533–1943)
Basal measures		
HOMA-IR	1.62 ± 0.96	(0.90–2.10)
HOMA-B	116.2 ± 45.1	(85.7–142.6)

*IRAS-FS* Insulin Resistance Atherosclerosis Family Study, *S*_*I*_ insulin sensitivity, *S*_*G*_ glucose effectiveness, *AIR* acute insulin response to glucose, *DI* disposition index, *HOMA-IR* homeostatic model assessment of insulin resistance, *HOMA-B* homeostatic model assessment of beta-cell function

### Associated metabolites in univariate models

The associations between 727 metabolites and glucose homeostasis traits were assessed using the general linear mixed model with adjustment for age, sex, BMI and pedigree structure. There were 79, 2, 58, 40, 45 and 19 metabolites significantly associated with S_I_, AIR, DI, S_G,_ HOMA-IR and HOMA-B, respectively (*P* < 6.8 × 10^–5^, See Table 2 and Supplementary Tables 4a-f for each glucose homeostasis trait).

**Table 2:** The most significantly associated metabolites (*P* < 6.8 × 10^–5^) in each super-pathway for glucose homeostasis traits

Trait	Super-pathway	Number of significant metabolites	Most significant metabolite	Sub-pathway	Beta ± SE	*P* value
S_I_	Amino Acid	19	Glycine	Glycine, Serine and Threonine Metabolism	0.54 ± 0.06	6.49E-17
	Lipid	48	1-stearoyl-2-dihomo-linolenoyl-GPC (18:0/20:3n3 or 6)*	Phospholipid Metabolism	− 0.33 ± 0.047	5.15E-12
	Carbohydrate	3	Pyruvate	Glycolysis, Gluconeogenesis, and Pyruvate Metabolism	− 0.28 ± 0.04	1.93E-09
	Cofactors and Vitamins	1	Quinolinate	Nicotinate and Nicotinamide Metabolism	− 0.15 ± 0.03	4.16E-06
	Nucleotide	2	Urate	Purine Metabolism, (Hypo)Xanthine/Inosine containing	− 0.44 ± 0.09	7.93E-07
	Peptide	6	Gamma-glutamylglycine	Gamma-glutamyl Amino Acid	0.31 ± 0.05	4.68E-10
AIR	Lipid	1	1-docosapentaenoyl-GPC (22:5n3)*	Lysolipid	− 4.42 ± 1.028	2.05E-05
	Carbohydrate	1	Glucose	Glycolysis, Gluconeogenesis, and Pyruvate Metabolism	− 36.59 ± 4.62	1.90E-14
DI	Carbohydrate	4	Glucose	Glycolysis, Gluconeogenesis, and Pyruvate Metabolism	− 51.03 ± 5.93	1.32E-16
	Lipid	37	Palmitate (16:0)	Long Chain Fatty Acid	− 16.65 ± 2.66	8.99E-10
	Amino Acid	12	3-methyl-2-oxobutyrate	Leucine, Isoleucine and Valine Metabolism	− 21.41 ± 4.14	3.57E-07
	Energy	1	Aconitate [cis or trans]	TCA Cycle	− 17.98 ± 3.80	2.91E-06
	Nucleotide	1	Urate	Purine Metabolism, (Hypo)Xanthine/Inosine containing	− 17.94 ± 3.64	1.18E-06
	Peptide	3	Leucylleucine	Dipeptide	− 2.76 ± 0.64	1.81E-05
S_G_	Lipid	35	Palmitate (16:0)	Long Chain Fatty Acid	− 0.008 ± 0.001	4.90E-09
	Amino Acid	4	Alpha-ketobutyrate	Methionine, Cysteine, SAM and Taurine Metabolism	− 0.003 ± 0.001	1.30E-05
	Carbohydrate	1	Mannose	Fructose, Mannose and Galactose Metabolism	− 0.006 ± 0.001	1.37E-06
HOMA-IR	Amino Acid	21	2-oxoarginine*	Urea cycle; Arginine and Proline Metabolism	0.21 ± 0.03	1.13E-11
	Carbohydrate	3	Pyruvate	Glycolysis, Gluconeogenesis, and Pyruvate Metabolism	0.39 ± 0.07	7.29E-09
	Cofactors and Vitamins	1	Quinolinate	Nicotinate and Nicotinamide Metabolism	0.26 ± 0.05	9.86E-09
	Lipid	16	1-(1-enyl-palmitoyl)-2-oleoyl-GPC (P-16:0/18:1)*	Plasmalogen	− 0.48 ± 0.07	4.53E-10
	Peptide	4	Gamma-glutamylvaline	Gamma-glutamyl Amino Acid	0.49 ± 0.08	4.13E-09
HOMA-B	Amino Acid	9	2-oxoarginine*	Urea cycle; Arginine and Proline Metabolism	0.13 ± 0.02	2.42E-09
	Cofactors and Vitamins	1	Quinolinate	Nicotinate and Nicotinamide Metabolism	0.14 ± 0.03	1.47E-05
	Lipid	8	1-stearoyl-2-dihomo-linolenoyl-GPC (18:0/20:3n3 or 6)*	Phospholipid Metabolism	0.24 ± 0.05	4.54E-07
	Peptide	1	Gamma-glutamylvaline	Gamma-glutamyl Amino Acid	0.29 ± 0.06	6.26E-07

^*^Named compounds identified from mass and fragmentation analysis but not yet confirmed with standards

Association analyses revealed that 19 amino acid, 3 carbohydrate, 1 cofactors and vitamins, 48 lipid, 2 nucleotide and 6 peptide metabolites were significantly associated with S_I_ (Table 2 and Supplementary Table 4a). Except for asparagine and three metabolites from glycine-serine-threonine metabolism (glycine, N-acetylglycine and serine), all other metabolites from the amino acid group were negatively associated with S_I_, including eight BCAA amino acid (4.75 × 10^–13^ < *P* < 3.76 × 10^–5^) and one lysine (2-aminoadipate, *P* = 2.02 × 10^–12^) metabolites (Supplementary Table 4a). The metabolites glycine and glutamate were most significantly positively (*β* = 0.54, *P* = 6.49 × 10^–17^) and negatively (*β* = -0.35 and *P* = 6.54 × 10^–16^) associated with S_I,_ respectively. Nine lipid metabolites were positively associated with S_I_ but the remaining 39 lipid metabolites were negatively associated with S_I_ (Supplementary Table 4a). Most significantly associated metabolites in diacylglycerol, long chain fatty acid, phospholipid, polyunsaturated fatty acid, and sphingolipid metabolism were negatively associated with S_I_ (Supplementary Table 4a).

Examination of AIR revealed significant association with two metabolites (Table 2 and Supplementary Table 4b). The most prominent metabolite associated with AIR was glucose (*β* = -36.59, *P* = 1.90 × 10^–14^).

Of the 58 metabolites associated with DI, two metabolites (glycine and 1,2-dilinoleoyl-GPC (18:2/18:2)) were positively associated with DI but the remaining 56 metabolites were negatively associated with DI (Table 2 and Supplementary Table 4c). Twelve amino acid and 37 lipid metabolites were predominantly associated with DI. Seven of the 24 BCAA metabolites analyzed were negatively associated with DI (7.53 × 10^–7^ < *P* < 5.82 × 10^–5^). For the lipid group, 13 metabolites in long chain fatty acid, 5 metabolites in diacylglycerol, 6 metabolites in phospholipid, 6 metabolites in polyunsaturated and 3 metabolites in sphingolipid sub-pathways were associated with DI (Supplementary Table 4c). As observed for AIR, glucose was most negatively associated with DI (*β* = -51.03, *P* = 1.32 × 10^–16^) (Supplementary Table 4c). Given that DI is calculated as the product of S_I_ and AIR, a remarkable number of metabolites overlapped between S_I_ and DI, i.e., 34 of the 58 metabolites associated with DI were shared with S_I_. Most of these shared metabolites were less significantly associated with DI than S_I_. Three metabolites in the long chain fatty acid sub-pathway (margarate (17:0), palmitate (16:0), and stearate (18:0)) are, however, more significantly negatively associated with DI than S_I_. Moreover, 10 of the 24 metabolites that were unique to DI were from the long chain fatty acid sub-pathway (Supplementary Table 4c).

The associated metabolites for S_G_ were mainly clustered into two groups*:* amino acid (4 metabolites) and lipid (35 metabolites) (Table 2 and Supplementary Table 4d). All 4 amino acid metabolites were negatively associated with S_G_ (1.30 × 10^–5^ < *P* < 6.29 × 10^–5^). Except for three metabolites (propionylglycine, 1-linoleoyl-2-linolenoyl-GPC (18:2/18:3) and 1,2-dilinoleoyl-GPC (18:2/18:2)) in the lipid group, all other metabolites (including 10 fatty acid derivatives, 12 long chain fatty acids, 3 phospholipids, 5 polyunsaturated fatty acids, 2 sphingolipid metabolites, etc.) were negatively associated with S_G_ (Supplementary Table 4d).

Metabolite associations for S_I_, DI and S_G_ generally showed a similar pattern: the metabolites from amino acid and lipid groups were predominantly negatively associated with these three dynamic measures of glucose homeostasis traits. Sixteen metabolites overlapped between S_I_ and S_G_ with 11 metabolites from the lipid super-pathway group, whereas 26 metabolites overlapped between DI and S_G_ with 22 metabolites from the lipid super-pathway group.

**Fig. 1:**
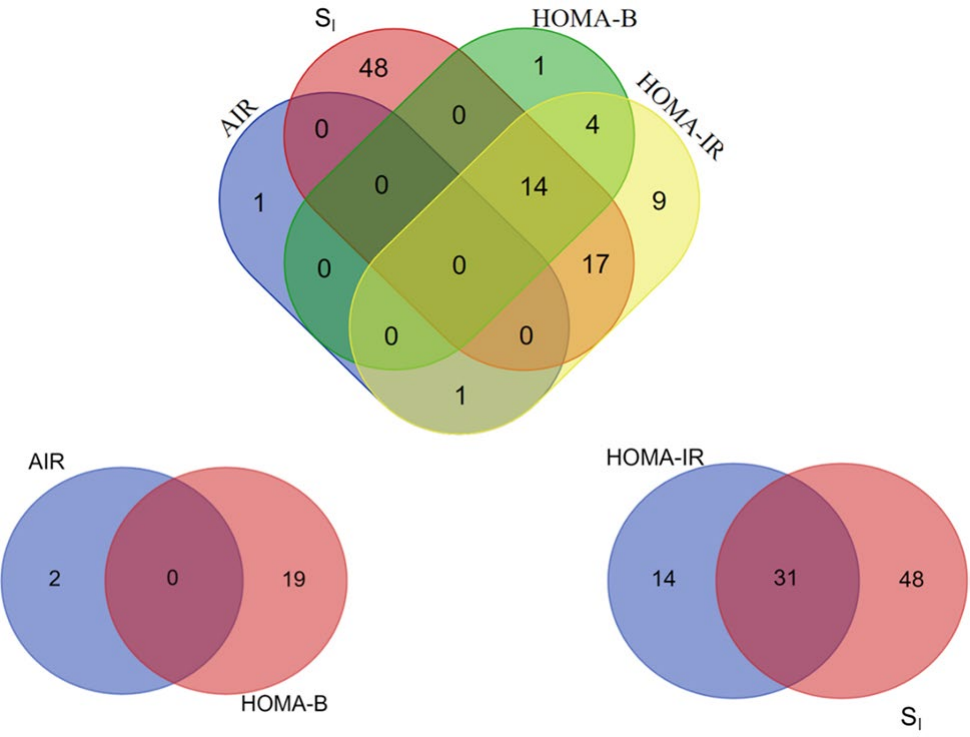
Overlap of significant metabolites between dynamic and basal measures of glucose homeostasis in African Americans. *AIR* acute insulin response, *HOMA-B* homeostatic model assessment of beta cell function, *HOMA-IR* homeostatic model assessment of insulin resistance, *S*_*I*_ insulin sensitivity

Association analyses revealed that 21 amino acid, 3 carbohydrate, 1 cofactors and vitamins, 16 lipid and 4 peptide metabolites were significantly associated with HOMA-IR (Table 2 and Supplementary Table 4e). Except for cysteine-glutathione disulfide and three metabolites from glycine-serine-threonine metabolism (glycine, N-acetylglycine and serine), all metabolites from the amino acid group were positively associated with HOMA-IR, including glutamate, five BCAA metabolites (8.95 × 10^–9^ < *P* < 3.31 × 10^–5^), one lysine metabolite (2-aminoadipate), five metabolites (2.68 × 10^–6^ < *P* < 6.51 × 10^–5^) in phenylalanine and tyrosine metabolism, and three metabolites (1.13 × 10^–11^ < *P* < 4.29 × 10^–8^) in the sub-pathway of urea cycle, arginine and proline metabolism (Supplementary Table 4e). Except two metabolites in plasmalogen sub-pathway, all other lipid metabolites were positively associated with HOMA-IR, including 4 from diacylglycerol, 1 from lysolipid, 7 from phospholipid, and 2 from plasmalogen metabolism sub-pathways (Supplementary Table 4e). Overall, 31 metabolites were significant associated with both HOMA-IR and S_I_ (Fig. 1).

The metabolites associated with HOMA-B were mainly clustered into two groups: the amino acid (9 metabolites) and lipid (8 metabolites) (Table 2 and Supplementary Table 4f). Three metabolites (glycine, *β* = -0.27, P = 2.6 × 10^–5^; N-acetylglycine, *β* = -0.13, P = 6.1 × 10^–7^; and serine, *β* = -0.40, P = 9.3 × 10^–6^) in the sub-pathway of glycine-serine-threonine metabolism were negatively associated with HOMA-B, but three metabolites (2.42 × 10^–9^ < *P* < 1.64 × 10^–7^) in the sub-pathway of urea cycle, arginine and proline metabolism were positively associated with HOMA-B. Except for 2-aminooctanoate, all other metabolites in the lipid group were positively associated with HOMA-B, including two diacylglycerol, four phospholipid, and one plasmalogen metabolites (Supplementary Table 4f). Overall, no metabolites were observed to overlap between HOMA-B and AIR (Fig. 1).

### Associated metabolites in regularized regression models

Overall, a smaller number of metabolites were selected by regularized regression models when compared with univariate models due to correlation among metabolites (Supplementary Table 4a-f and 5). The largest difference in the number of selected metabolites between the two models was observed for S_I_ (Supplementary Table 4a). The pairwise correlation heat map for metabolites associated with S_I_ in univariate models shows that metabolites within each of the sub-pathways (BCAA, diacylglycerol, long-chain fatty acid, lysolipid, phospholipid, sphingolipid, and gamma-glutamyl amino acid metabolisms) were positively correlated with each other (Supplementary Fig. 1). All the 15 metabolites associated with S_I_ in the regularized model overlapped those selected by univariate models (Supplementary Table 4a) and the metabolite glycine accounted for 11.4% of S_I_ residual variance. Two (glucose and 1-docosapentaenoyl-GPC (22:5n3)) of the 7 metabolites associated with AIR in the regularized model overlapped those associated in the univariate model (Supplementary Table 4b) and glucose accounted for 9.4% of AIR residual variance. Ten of the 12 metabolites associated with DI in the regularized model overlapped those associated in the univariate model (Supplementary Table 4c) and the top three metabolites (glucose, palmitate and leucine) accounted for 19.2% of DI residual variance. Four of the 6 metabolites associated with S_G_ in the regularized model overlapped those associated in the univariate model (Supplementary Table 4d), and the metabolite palmitate accounted for 6.0% of S_G_ residual variance. Ten of the 11 metabolites associated with HOMA-IR in the regularized model overlapped those associated in the univariate model (Supplementary Table 4e), and the metabolite 2-oxoarginine accounted for 8.1% of HOMA-IR residual variance. Eight of the 10 metabolites associated with HOMA-B in the regularized model overlapped those associated in the univariate model (Supplementary Table 4f), and the metabolite 2-oxoarginine accounted for 5.9% of HOMA-B residual variance.

The regularized models including a smaller set of metabolites for S_I,_ AIR, DI, S_G,_ and HOMA-B explained a similar proportion of residual variance (R^2^_adj_ = 0.31, 0.20, 0.23, 0.14 and 0.21, respectively) compared with the univariate models including all associated metabolites (R^2^_adj_ = 0.33, 0.16, 0.26, 0.18 and 0.17, respectively) except for HOMA-IR (R^2^_adj_ = 0.22 for regularized model; R^2^_adj_ = 0.41 for univariate model) (Supplementary Table 5).

### Comparison of metabolite associations with glucose homeostasis traits between African Americans and Mexican Americans

African Americans were more insulin resistant (low S_I_) but had better first phase insulin response (AIR) than Mexican Americans in the IRAS-FS (Supplementary Table 2). We explored whether there were any metabolites that were differentially associated with these six glucose homeostasis traits between these two populations. For most of the metabolites analyzed, no significant differences in associations with these glucose homeostasis traits were observed (Supplementary Table 6). However, the effect sizes of glutamate on S_I_ was significantly different for S_I_ (β = − 0.35 and -0.12 in African Americans and Mexican Americans, respectively; *P* = 4.5 × 10^–6^, Table 3 and Supplementary Table 6). Glutamate was also differentially associated with DI, S_G_ and HOMA-IR at nominal significance with larger effects observed in African Americans than in Mexican Americans (Table 3 and Supplementary Table 6).

**Table 3:** Differential glutamate associations with glucose homeostasis traits between African Americans and Mexican Americans

Glucose homeostasis traits	African Americans (AA, N = 571)			Mexican Americans (MA, N = 1,111)			*P* value between AA and MA
	Beta	Stderr	*P* value	Beta	Stderr	*P* value	
S_I_	− 0.352	0.045	6.54E-14	− 0.117	0.024	7.85E-07	4.55E-06
AIR	2.093	1.520	1.69E-01	2.303	0.590	1.04E-04	8.98E-01
DI	− 8.498	1.937	1.44E-05	− 0.356	0.853	6.77E-01	1.20E-04
S_G_	− 0.003	0.0009	1.40E-03	0.0001	0.0005	8.00E-01	2.88E-03
HOMA-IR	0.423	0.065	2.28E-10	0.195	0.026	1.96E-13	1.17E-03
HOMA-B	0.172	0.047	2.44E-04	0.090	0.019	1.54E-06	1.02E-01

The association results for IRAF-FS Mexican Americans were published previously (Palmer et al., 2018)

## Discussion

We performed a comprehensive assessment of the signature of metabolomics associated with dynamic and basal measures of glucose homeostasis traits in 571 non-diabetic African Americans. We applied both univariate and regularized regression models to identify metabolites that were significantly associated with six glucose homeostasis traits (S_I_, AIR, DI, S_G_, HOMA-IR and HOMA-B). The univariate models identified multiple significantly correlated metabolites within the same sub-pathway; however, the regularized regression models identified the most important metabolites within each sub-pathway. Overall, the metabolites involved in amino acid metabolism (BCAA metabolism; glycine, serine and threonine metabolism; lysine metabolism; methionine, cysteine, SAM and taurine metabolism; glutamate metabolism; urea cycle, arginine and proline metabolism), carbohydrate metabolism (glycolysis, gluconeogenesis, and pyruvate metabolism) and lipid metabolism (diacylglycerol and phospholipid metabolism) were associated with both basal and dynamic measures of glucose homeostasis traits. In addition, we observed that glutamate affects glucose homeostasis traits more strongly in African Americans than in Mexican Americans.

Decades of epidemiology studies have established that elevated levels of circulating BCAAs and their metabolites are associated with insulin resistance and type 2 diabetes risk in populations of European ancestry (Felig et al., 1969; Gall et al., 2010; Guasch-Ferre et al., 2016; Huffman et al., 2009; Menni et al., 2013; Newgard et al., 2009; Shah et al., 2012; Stancakova et al., 2012; Tillin et al., 2015; Wang et al., 2011; Wurtz et al., 2012a, 2012b, 2013). These associations were recently confirmed in populations of Asian (Arany & Neinast, 2018; Chen et al., 2016, 2019; Tai et al., 2010; Takashina et al., 2016; Tillin et al., 2015), African (Chen et al., 2022) and Mexican (Lee et al., 2016; Palmer et al., 2015, 2018) ancestries. In this study, we observed in African Americans that increased levels of BCAA-related metabolites (isoleucine, 3-methyl-2-oxovalerate and 3-hydroxy-2-ethylpropionate in isoleucine metabolism; leucine, 4-methyl-2-oxopentanoate and isovalerylcarnitine in leucine metabolism; valine, 3-methyl-2-oxobutyrate and 3-hydroxyisobutyrate in valine metabolism; Supplementary Table 4a-f) were positively associated with basal measures of HOMA-IR but negatively associated with dynamic measures of S_I_, DI and S_G_. Isoleucine and valine were recently demonstrated to be involved in reprograming liver and adipose metabolism to reduce hepatic insulin sensitivity and ketogenesis and energy expenditure and mediate the adverse metabolic effects of BCAAs (Yu et al., 2021).

Circulating levels of glycine were lower in obese (Felig et al., 1969; Okekunle et al., 2017; Takashina et al., 2016; Yan et al., 2012) and insulin-resistant individuals (Ejaz et al., 2016; Gall et al., 2010; Newgard et al., 2009; Takashina et al., 2016) as compared to healthy individuals in populations of European and East Asian ancestries. Plasma glycine levels were also negatively associated with fasting glucose levels and type 2 diabetes risk (Chen et al., 2022; Ferrannini et al., 2013; Floegel et al., 2013; Guasch-Ferre et al., 2016; Newgard et al., 2009; Palmer et al., 2015; Svingen et al., 2016; Vangipurapu et al., 2019; Walford et al., 2016; Wang-Sattler et al., 2012). In concordance with the previous findings, we observed that the metabolites (glycine, N-acetylglycine or serine; Supplementary Table 4a-f) in the glycine, serine and threonine metabolism sub-pathway were positively associated with dynamic measures of S_I_ and DI but negatively associated with basal measures of HOMA-IR and HOMA-B in IRAS-FS African Americans. The metabolite glycine also explained the largest residual variance (11.4%) of S_I_ in IRAS-FS African Americans. Glycine plasma concentration is tightly regulated by glucagon, which is a major regulator of hepatic glycine metabolism. Elevated plasma glucagon levels could drive increased glycine degradation in insulin resistant states (Alves et al., 2019). In addition, impaired hepatic BCAA metabolism in obesity was shown to contribute to the decrease in glycine circulating concentration; however, the underlying cellular mechanisms still remain to be fully elucidated (Alves et al., 2019).

Lysine and its metabolite 2-aminoadipate were associated with increased type 2 diabetes risk in populations of both European and Asian ancestries (Chen et al., 2019; Takashina et al., 2016; Wang et al., 2013). The metabolite 2-aminoadipate was negatively associated with dynamic measures of S_I_ and DI but positively associated with basal measures of HOMA-IR in IRAS-FS African Americans (Supplementary Table 4a-f). The in vitro studies suggest that 2-aminoadipate had an effect on insulin secretion in pancreatic β-cells and isolated islets (Wang et al., 2013) and the positive associations between 2-aminoadipate levels and type 2 diabetes risk in human may be due to the development of insulin resistance secondary to chronic hyperinsulinemia (Newgard, 2017; Wang et al., 2013).

The metabolite 2-hydroxybutyrate in the sub-pathway of methionine, cysteine, SAM and taurine metabolism was an early biomarker of insulin resistance and glucose intolerance in a nondiabetic population of European ancestry (Gall et al., 2010) and its levels was elevated in type 2 diabetes patients compared with healthy controls in populations of Asian ancestry (Li et al., 2009). Consistent with our previous findings in IRAS-FS Mexican Americans (Palmer et al., 2018), this metabolite was negatively associated with dynamic measures of S_I_, DI and S_G_ in IRAS-FS African Americans (Supplementary Table 4a-f). The metabolite 2-hydroxybutyrate is converted from alpha-ketobutyrate when the NADH/NAD^+^ ratio is elevated in high lipid oxidations of insulin resistant states (Gall et al., 2010). The NMD supplementation is recently proven to be effective in recovering insulin sensitivity in prediabetic women (Yoshino et al., 2021).

Plasma glutamate levels were differentially associated with glucose homeostasis traits between African Americans and Mexican Americans in IRAS-FS with larger effects (negatively associated with dynamic measures of S_I_, DI and S_G_ but positively associated with measures of HOMA-IR) observed in African Americans than in Mexican Americans. Oral glutamate supplementation was previously shown to impair insulin sensitivity in a short-term dietary intervention (Chevassus et al., 2002). Increased circulating levels of glutamate were previously associated with insulin resistance (Chen et al., 2022; Cheng et al., 2012; Newgard et al., 2009; Vangipurapu et al., 2019) and type 2 diabetes risk in populations of European (Chen et al., 2019; Cheng et al., 2012; Ferrannini et al., 2013; Liu et al., 2019; Stancakova et al., 2012; Vangipurapu et al., 2019), Asian (Chen et al., 2019; Takashina et al., 2016) and African (Chen et al., 2022). ancestries. Glutamate stimulated glucagon release from pancreatic α-cells (Adrover et al., 2015) and increased transamination of pyruvate to alanine, a strong promoter of gluconeogenesis (Newgard et al., 2009). These mechanisms may partially explain the increased type 2 diabetes risk associated with circulating glutamate levels. Populations of African ancestry are generally more susceptible to insulin resistance than populations of other ancestries (Meigs et al., 2014); however, the underlying physiological mechanisms of increased glutamate sensitivity on glucose homeostasis traits in African Americans merits further investigations.

The amino acid sub-pathway of urea cycle, arginine and proline metabolism was consistently enriched for dynamic measures of S_I_ and basal measures of HOMA-IR and HOMA-B in IRAS-FS African Americans (Supplementary Table 4a-f). The metabolite 2-oxoarginine in this pathway was negatively associated with S_I_, but most significantly positively associated with measures of HOMA-IR and HOMA-B levels. It also explained the largest residual variance of HOMA-IR (8.1%) and HOMA-B (5.9%). Similarly, the metabolite arginine in this pathway was negatively associated with S_I_ but positively associated with HOMA-IR and HOMA-B levels (Supplementary Table 4a-f). Increased plasma levels of 2-oxoarginine and arginine were positively associated with HOMA-B measures in IRAS-FS Mexican Americans (Palmer et al., 2018), and increased circulating arginine levels were associated with increased type 2 diabetes risk in populations of European (Guasch-Ferre et al., 2016) and African (Chen et al., 2022) ancestries. The metabolite 2-oxoarginine is a guanidino compound metabolite of arginine catabolism. Arginine promoted insulin secretion (Sener et al., 2000). However, the underlying mechanisms of arginine metabolites regulating glucose homeostasis traits remains unclear.

The carbohydrate sub-pathway of glycolysis, gluconeogenesis, and pyruvate metabolism was consistently enriched for dynamic measures of S_I_, AIR, DI and S_G_, and basal measures of HOMA-IR in IRAS-FS African Americans (Supplementary Table 4a-f). Several metabolites (glucose, fructose, mannose, lactate, pyruvate, mannose or ribonate) were negatively associated with S_I_, AIR, DI, S_G_, but positively associated with HOMA-IR, consistent with reported higher levels of these metabolites in individuals with type 2 diabetes than control subjects of European ancestry (Drogan et al., 2015; Floegel et al., 2013; Guasch-Ferre et al., 2016; Padberg et al. 2014; Wurtz et al., 2012a, 2012b). The metabolite glucose explained the largest residual variance of AIR (9.4%) and DI (10.9%) and confirmed its important role of regulating these glucose homeostasis traits.

Circulating medium- and long-chain fatty acids were elevated in prediabetes and type 2 diabetes patients in populations of European and Asian ancestries (Gall et al., 2010; Li et al., 2009; Menni et al., 2013). Plasma levels of long-chain fatty acids were negatively associated with dynamic measures of S_I_, DI and S_G_ in the IRAS-FS Mexican Americans (Palmer et al., 2018). We observed a similar pattern of increased levels of palmitate (C16), margarate (C17) and stearate (C18) associated with reduced S_I_ in IRAS-FS African Americans (Supplementary Table 4a-f). In addition, more long-chain fatty acids species (n = 13, Supplementary Table 4c) were inversely associated with DI in IRAS-FS African Americans. The metabolite palmitate also explained a substantial portion of residual variance of DI (5.8%) and S_G_ (6.0%). The increased plasma medium- and long-chain fatty acid levels may be biomarkers of increased adipose fatty acid releases and/or reduced fatty acid oxidations of insulin resistant states.

Our study has the following strengths. It included the largest sample size of African Americans in a semi-untargeted metabolomic study with 727 “known” plasma metabolites assayed. While most studies have fasting glucose and insulin measures only (Chen et al., 2022; Rebholz et al., 2018; Yu et al., 2019), we analyzed both basal and dynamic glucose homeostasis, of which dynamic measures could be considered physiologically more proximal to pathogenic components of type 2 diabetes and provide discrete insights into the pathogenesis of type 2 diabetes (Palmer et al., 2018). The comparison of metabolomic profiles between African Americans and Mexican Americans in IRAS-FS allows identification of differentially associated metabolites, while other studies only reported associations adjusted for ancestry (Chen et al., 2022; Rebholz et al., 2018). We used regularized models to identify key metabolites associated with these glucose homeostasis traits, which could partially overcome the bias derived from high correlation of metabolites in the same metabolic sub-pathway; and finally, substantial proportions of residual variance in these glucose homeostasis traits (ranging from 16 to 41% in univariate models) have been explained by these metabolites even after adjustment for the effect of BMI (Supplementary Table 5). However, the current study is observational in nature, which precludes assessment of causality between metabolites and these glucose homeostasis traits, thus unable to determine which participants may develop diabetes in the future.

In summary, we confirmed in a representative population of African ancestry that increased plasma metabolite levels of BCAA and their metabolites, 2-aminoadipate, 2-hydroxybutyrate, glutamate, arginine and its metabolites, carbohydrate metabolites, and median- and long-chain fatty acids are associated with insulin resistance, while increased plasma metabolite levels in the glycine, serine and threonine metabolic sub-pathway were associated with insulin sensitivity. We also observed a differential ancestral effect of glutamate on glucose homeostasis with much stronger effect observed in African Americans than those observed in Mexican Americans. Overall, this study suggests that these metabolites may be useful biomarkers in the identification of prediabetes individuals at risk of type 2 diabetes in African Americans, which warrant further studies. Our findings also extend the scientific literature on the role of these metabolites in the etiology of insulin resistance and impaired insulin secretion and highlight the need for additional comprehensive metabolomic studies in well-characterized multiethnic cohorts.

## Supplementary Information

Below is the link to the electronic supplementary material.

Supplementary file1 (DOCX 551 kb)

Supplementary file2 (XLSX 486 kb)

## Data Availability

The datasets generated during and/or analyzed during the current study are available from the corresponding author on reasonable request.

## Abbreviations

AIR: Acute insulin response
BCAA: Branched-chain amino acids
COMETS: Consortium of Metabolomics Studies
DI: Disposition index
FSIGT: Frequently sampled intravenous glucose tolerance test
HOMA-IR: Homeostatic model assessment of insulin resistance
HOMA-B: Homeostatic model assessment of beta cell function
IRAS-FS: Insulin Resistance Atherosclerosis Family Study
MS: Mass spectrometry
S_G_: Glucose effectiveness
S_I_: Insulin sensitivity
