# Supplementary material for: Metabolomic profiling of glucose homeostasis in African Americans: the Insulin Resistance Atherosclerosis Family Study (IRAS-FS)
Source: Metabolomics. 2023 Apr 2;19(4):35. doi: 10.1007/s11306-023-01984-1 (PMC10068644; doi:10.1007/s11306-023-01984-1)

**Supplementary document**

**Metabolomic profiling of glucose homeostasis in African Americans: The Insulin Resistance Atherosclerosis Family Study (IRASFS)**

Hayrettin Okut^1,2,^*, Yingchang Lu^3,^*, Nicholette D. Palmer^1,4^, Yii-Der Ida Chen^5^, Kent D. Taylor^5^, Jill M. Norris^6^, Carlos Lorenzo^7^, Jerome I. Rotter^5^, Carl D. Langefeld^8^, Lynne E. Wagenknecht^9^, Donald W. Bowden^1,4,10^, Maggie C.Y. Ng^1,3^

^1^Center for Precision Medicine, Wake Forest School of Medicine, Winston-Salem, NC, USA;

^2^Department of Population Health, University of Kansas School of Medicine-Wichita, Wichita, KS, USA;

^3^Vanderbilt Genetics Institute, Division of Genetic Medicine, Vanderbilt University Medical Center, Nashville, TN, USA;

^4^Department of Biochemistry, Wake Forest School of Medicine, Winston-Salem, NC, USA;

^5^The Institute for Translational Genomics and Population Sciences, Department of Pediatrics, The Lundquist Institute for Biomedical Innovation at Harbor-UCLA Medical Center, Torrance, CA USA;

^6^Departments of Epidemiology, Colorado School of Public Health, University of Colorado Denver, Aurora, CO, USA;

^7^Department of Medicine, University of Texas Health Science Center, San Antonio, TX, USA;

^8^Department of Biostatistical Sciences, Wake Forest School of Medicine, Winston-Salem, NC, USA;

^9^Division of Public Health Sciences, Wake Forest School of Medicine, Winston-Salem, NC, USA;

^10^Department of Internal Medicine, Wake Forest School of Medicine, Winston-Salem, NC, USA;

* these authors contribute equally to this manuscript

Correspondence:

Maggie C.Y. Ng, Vanderbilt Genetics Institute, Division of Genetic Medicine, Vanderbilt University Medical Center, Nashville, TN 37232, USA

Email: maggie.ng@vumc.org

**Supplementary Figure 1. Heat map of metabolites organized by super-pathways and sub-pathways which were significantly associated with S_I_ from univariate models (A) and from elastic net models (B) for IRAS-FS African Americans.**


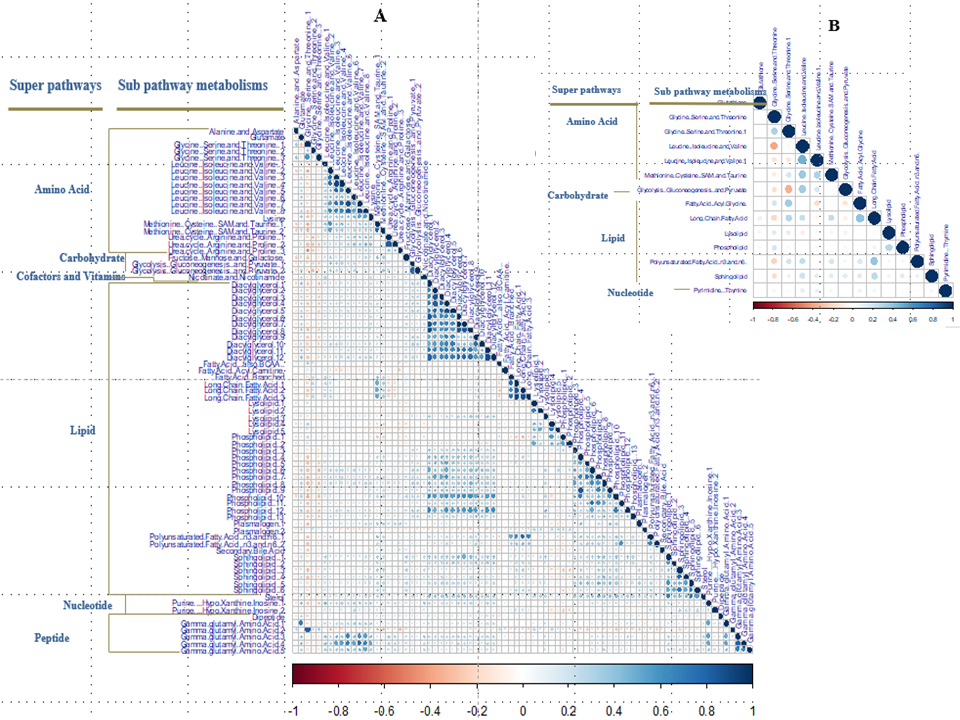

Supplement: Supplementary file 1 — Supplementary file1 (DOCX 551 kb) [file 11306_2023_1984_MOESM1_ESM.docx]
